# A Biodegradable Flexible Micro/Nano-Structured Porous Hemostatic Dental Sponge

**DOI:** 10.3390/nano12193436

**Published:** 2022-09-30

**Authors:** Simin Sharifi, Solmaz Maleki Dizaj, Elham Ahmadian, Alireza Karimpour, Abdollah Maleki, Mohammad Yousef Memar, Mohammad Ali Ghavimi, Elaheh Dalir Abdolahinia, Khang Wen Goh

**Affiliations:** 1 Dental and Periodontal Research Center, Tabriz University of Medical Sciences, Tabriz 5166/15731, Iran; 2 Department of Dental Biomaterials, Faculty of Dentistry, Tabriz University of Medical Sciences, Tabriz 5166/15731, Iran; 3 Kidney Research Center, Tabriz University of Medical Sciences, Tabriz 5166/15731, Iran; 4 Kimia Pajuhesh Nanofarnam Compony, Tabriz Medical Equipment Technology Incubator Center, Tabriz University of Medical Sciences, Tabriz 5166/15731, Iran; 5 Non-Destructive Testing Lab, Department of Mechanical Engineering, Amirkabir University of Technology, 424 Hafez Ave, Tehran 15914, Iran; 6 Infectious and Tropical Diseases Research Centre, Tabriz University of Medical Sciences, Tabriz 5166/15731, Iran; 7 Department of Oral and Maxillofacial Surgery, Faculty of Dentistry, Tabriz University of Medical Sciences, Tabriz 5166/15731, Iran; 8 Research Center for Pharmaceutical Nanotechnology, Tabriz University of Medical Sciences, Tabriz 5166/15731, Iran; 9 Faculty of Data Sciences and Information Technology, INTI International University, Nilai 78100, Malaysia

**Keywords:** dentistry sponge, gelatin, hemostatic effect, nanomedicines, nanotechnology

## Abstract

A biodegradable micro/nano-structured porous hemostatic gelatin-based sponge as a dentistry surgery foam was prepared using a freeze-drying method. In vitro function evaluation tests were performed to ensure its hemostatic effect. Biocompatibility tests were also performed to show the compatibility of the sponge on human fetal foreskin fibroblasts (HFFF2) cells and red blood cells (RBCs). Then, 10 patients who required the extraction of two teeth were selected, and after teeth extraction, for dressing, the produced sponge was placed in one of the extracavities while a commercial sponge was placed in the cavity in the other tooth as a control. The total weight of the absorbed blood in each group was compared. The results showed a porous structure with micrometric and nanometric pores, flexibility, a two-week range for degradation, and an ability to absorb blood 35 times its weight in vitro. The prepared sponge showed lower blood clotting times (BCTs) (243.33 ± 2.35 s) and a lower blood clotting index (BCI) (10.67 ± 0.004%) compared to two commercial sponges that displayed its ability for faster coagulation and good hemostatic function. It also had no toxic effects on the HFFF2 cells and RBCs. The clinical assessment showed a better ability of blood absorption for the produced sponge (*p*-value = 0.0015). The sponge is recommended for use in dental surgeries because of its outstanding abilities.

## 1. Introduction

Blood clot formation and hemostasis are considered essential principles of the surgery. Even in simple extractions, rapid clotting prevents infection and commences the healing and tissue repair process [1]. There are numerous hemostatic materials for different clinical uses, which can be categorized as non-absorbable and absorbable materials. Non-absorbable hemostatic materials may lead to infection and needs to be removed from the bleeding site. These issues restrict its usage in clinical practice, especially in emergency applications [2]. Absorbable materials that help hemostasis and may be used to accelerate healing are advantageous in surgery. In controlling bleeding, proper handling is vital and an appropriate amount should only be used, although it is expected that the hemostat dissolves promptly. A dry local hemostat may be used to absorb substances several times its weight and can expand postoperatively [3]. Thus, when using an absorbable hemostatic agent near or on neural or bony spaces, the least possible amount should be left when hemostasis is performed. Minimum inflammation with no blockade of healing or strong foreign body reactions is desirable when using local hemostats [3,4].

Sponge-type materials show a better hemostatic result than other common dressings such as surgical gauzes. Due to the porous structure, the amount of blood absorbed by porous hemostatic sponges is larger than normal ones, which can decrease the occurrence of postoperative concerns, such as hematoma [5,6]. The micro/nano-structured porous sponges are also at the core of attention for applications as hemostatic agents. The micrometric pores can use for trapping red blood cells (RBCs), and nanometric pores may help improve water sorption (plasma sorption) [7]. Nanometer-scale pores on the surface of the sponge can also lead to surface roughness [8].

The absorbable hemostatic polymers that are presently applied mostly include chitosan, collagen, gelatin, sodium alginate, and cellulose [9]. Many polymer-based sponges have been advanced to attain hemostatic function and wound healing. Chitosan is known as one of the main polymers in hemostatic applications due to its anti-inflammatory possessions, biocompatibility, and good in vivo degradation. However, chitosan has low solubility in water and then has poor adsorption, which makes it a weak local hemostatic agent [10]. Two forms of cellulose sponge including oxidized cellulose and oxidized regenerated cellulose, are also available as hemostatic sponges [11]. Collagen is extensively applied in tissue engineering. It also shows good hemostatic effects under natural conditions, which converts it into a good wound hemostatic material [2,4]. Gelatin, as a natural water-soluble biocompatible macromolecule, is one type of protein produced by the partial hydrolysis of native collagen. It is vastly used for the production of hemostatic surgery sponges. It is considered to contribute to macrophage activation and has a great hemostatic effects. Gelatin is, in particular, more convenient compared with commercial collagen since the preparation of concentrated collagen solutions is very difficult when using native collagen; therefore, using gelatin is more economical compared with collagen [12,13]. Gelatin sponges can trap platelets and red blood cells in their matrix [14]. Some marketed hemostatic gelatin-based sponges are summarized in Table 1.

The recognized method to control dental post-extraction bleeding is to use a sterile gauze [15]. However, the bleeding control of dental post-extraction is influenced by some aspects, including the site of bleeding, wound size, the extent of bleeding, accessibility of the bleeding site, the timing of bleeding, and the condition of the patient (any systemic disease, such as cirrhosis, that can affect bleeding and coagulation) [16,17,18]. Dental surgery sponges, with the generic name of Gelfoam, are used for wound infection and secondary bleeding prophylaxis after extractions [19]. They are well-tolerated absorbable products that may be used for safe wound treatments with respect to wounded alveoli and hemostasis with respect to cavities [20]. A good dental sponge must have more rapid primary wound hemostasis, uniform and complete high absorption capacities, biocompatibility, good clot stabilization, and be completely absorbable within four weeks. Advantages of using surgery sponges in dentistry include improved healing processes, not requiring secondary surgeries for sponge removal, and saving time because of high hemostatic capacities [21,22,23]. The use of dental surgery sponges can also reduce the incidence of postoperative issues with respect to dental extraction, such as pain, swelling, the occurrence of dry sockets, and inflammation [24,25,26].

**Table 1:** Some marketed hemostatic gelatin-based sponges.

Sponge Brand	Producer	Applications	Ref.
Gelfoam^®^	Pharmacia & Upjohn Co., Kalamazoo, MI, USA	Dental	[14]
Gelita-Spon^®^	Invotec International, Inc, Jacksonville, FL, USA	Dental, nasal, sinus surgery, or any other surgical procedure	[14]
SPONGOSTAN ^TM^	Johnson & Johnson, Ferrosan, Søborg, Denmark	Dental	[27]
SURGIFOAM^®^	Ethicon, Somerville, NJ, USA	Dental, Oral Surgery	[27]
Gelatamp	Roeko-Coltène/Whaledent, Langenau, Germany	Dental, Oral Surgery	[28]

The goal of this study was to assess a biodegradable micro/nano-structured porous hemostatic gelatin-based sponge as a dental surgery foam via the freeze-drying method.

## 2. Materials and Methods

### 2.1. Preparation of Sponge

**Figure 1:**
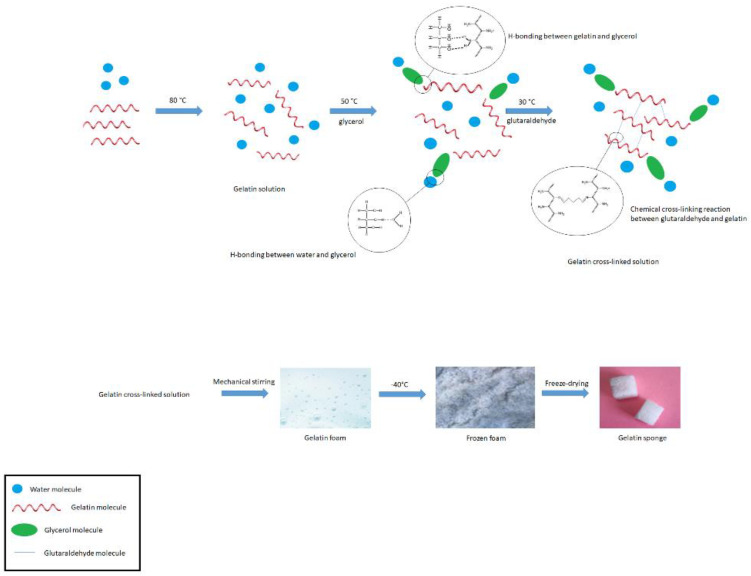
A scheme for the reaction steps.

Gelatin solution was prepared with a weight ratio of 10 to 90 of gelatin (G9391-Sigma Aldrich, St. Louis, MO, USA) and distilled water (Pars Company, Dastgerd, Iran) at a temperature of 80 °C on a hot plate. Then, the temperature decreased to 50 °C and two percent (*w*/*w*%) of glycerol (Millipore Merck-EMSURE grade, code 104092) was added to the solution. It was then stirred on a hot plate at 30 °C and one percent (*w*/*w*%) of glutaraldehyde (G6257-Sigma Aldrich) as a crosslinking agent was added; stirring continued. The resulting solution was poured into a mechanical stirrer and stirred for one hour. The resulting foam was then poured into rectangular molds and placed in a freezer at −40 °C for 24 h. The frozen material was placed in a freeze-dryer for 24 h and dried. The final dried product (gelatin sponge) was cut into the desired dimensions. The cut material was packaged and sterilized by gamma rays (25 kgry). Figure 1 shows a scheme for the reaction steps.

### 2.2. Physicochemical and Physicomechanical Characterization

#### 2.2.1. Sponge Structure and Morphology

Samples (gelatin sponge) were located on a special scanning electron microscope plate (FE-SEM type S-1640 HITACHI company (Tokyo, Japan)) and coated with gold. The image was taken at proper magnification (100×). Energy-dispersive X-ray (EDX) analysis was also performed to show the elements present in the samples.

#### 2.2.2. Identification of the Functional Groups

The identification of functional groups in the samples of gelatin and the final product (gelatin sponge) was performed by FT infrared spectroscopy (FTIR) in the range of 4000–400 cm^−1^, using BRUKER spectrophotometer model Tensor 27 IR and potassium bromide powder as the reference material.

#### 2.2.3. Identification of Crystallinity State

XRD patterns were taken for the gelatin and the final product at room temperature. The samples were exposed to an X-ray diffractometer (Cu Kα Xray radiation source Shimadzu 6000 (Shimadzu, Japan)) and a wavelength of 5405/1 Å, a voltage of 40 kV, and a current of 30 mA, and their patterns were recorded by an angle recorder. Two thetas were recorded at 5 to 60 degrees.

#### 2.2.4. Tensile Strength Determination

The Hounsfield H5K-(UK) apparatus was utilized to assess the tensile strength of the samples. The stretching rate of the device was set at one millimeter per minute. The constructed sponges (n = 3) were pulled to a cross-section of 10 × 2 mm with the help of two clamps.

#### 2.2.5. The Specific Surface Area, Total Pore Volume, and Porosity

Brunauer–Emmett–Teller (Osaka, Japan) analysis was applied to determine the specific surface area, total pore volume, and the porosity for the prepared sponge. The volume of absorbed nitrogen gas by the material was measured by gradually increasing the gas pressure. Then, with a gradual decrease in gas pressure, the amount of desorbed gas by the material was measured at a constant temperature.

#### 2.2.6. Volume Expansion

To predict the increase in the volume of sponge material at the site of the extracted tooth cavity, the volume expansion test was evaluated in vitro. In brief, the produced sponges (n = 3) were cut into dimensions of 10 × 10 × 10 mm. The initial volume of each sample was recorded (V_1_). Each sample was then placed separately into 50 mL balloons containing 25 mL of phosphate buffer (PBS) and incubated at 37 °C for one hour. When the samples reached the full volume (ensuring the absence of air bubbles), they were gently removed from the solution and placed inside a graduated cylinder containing distilled water of a specified volume. The amount of increased volume was recorded in the graduated cylinder (V_2_) and the expansion rate was evaluated as follows.

Volume expansion rate = V_2_/V_1_

#### 2.2.7. Degradation Test

To perform this test, sponges were cut in dimensions of 10 × 10 × 10 mm. The weight of each sample was recorded (W_0_). The samples were placed in 50 mL balloons and then PBS (25 mL) was added. Samples were incubated at 37 ° C for 4 weeks, and the weight of dried materials was noted for each week (W_W_1, W_W_2, W_W_3, and W_W_4). Finally, the percentage of material degradation was obtained as follows.

Substance degradation percentage= (W_0_ − W_w_/W_0_) × 100

### 2.3. Biocompatibility Assay

#### 2.3.1. Cellular Cytotoxicity

Cytotoxicity test was performed according to the ISO 10993-5 standard. Cytotoxicities (for 3 and 5 days) for the produced sponge were performed on human fibroblast cells (HFFF2).

One of the methods for assessing cytotoxicity is to measure cell viability by MTT or (3-(4,5-dimethylthiazol-2-yl)-2,5-diphenyltetrazolium bromide. For the test, HFFF2 cells were seeded in 96-well plates containing an RPIMI medium supplemented with 10% FBS. Sample pieces were placed in the center of each well, and cells were incubated for different time points (24, 48, and 72 h). Untreated cells were considered as negative controls. After the incubation periods, cells (containing samples/control cells) were incubated with 150 μL fresh medium with 50 μL MTT solutions (2 mg/mL in PBS) for 4 h at 37 °C. Then, the MTT-encompassing medium was detached, and a mixture of 200 μL DMSO and 25 μL Sorenson’s glycine buffer (0.1 M glycine, 0.1 M NaCl, pH 10.5) was added to each well; the cells were incubated once more for 30 min at 37 °C. The wells without cells were considered blanks. The absorbance of plates was measured at 570 nm by a microplate reader. Cell viability was read as follows.

$$\mathrm{Cell}\;\mathrm{Viability}\;(\%)=\frac{\mathrm{OD}\;\mathrm{test}-\mathrm{OD}\;\mathrm{blank}}{\mathrm{OD}\;\mathrm{control}-\mathrm{OD}\;\mathrm{blank}}\times 100$$

#### 2.3.2. Hemolysis Test

This test was performed on red blood cells by the ISO10993-4 standard. Accordingly, the percentage of hemolysis below two percent (2%) is considered a biocompatible substance. Conscious consent was received from a participant to provide a blood sample.

To perform the test, 2 mL of fresh whole blood was poured into a hematocrit tube. It was then centrifuged at 3000 rpm for 5 min and washed three times with PBS solution (PH = 7.4). The resulting red blood cells were diluted from 1 to 10. Then, 0.5 mL of the diluted sample was poured into a test tube, and samples of the fabricated sponge (3 pieces) were thrown into the tubes. The PBS solution was considered a negative control, and distilled water was considered a positive control. The tubes were incubated for 2 h at 37 °C. The morphology of red blood cells was tested under a light microscope at 100×. All tubes were centrifuged at 5000 rpm for 5 min, and then 200 μL of the top solution of each tube was transferred to the microplate’s housings. The results of RBCs lysis were read at 540 nm (OD) and calculated via the following formula.

$$\mathrm{Hemolysis}\;\mathrm{Rate}\;(\%)=\frac{\mathrm{OD}\left(s\right)-\;\mathrm{OD}\left(-\right)}{\mathrm{OD}\left(+\right)-\;\mathrm{OD}\left(-\right)}\times 100$$

### 2.4. Function Evaluation Tests

#### 2.4.1. Blood Absorption Test and Swelling Percentage

To test the amount of blood absorption, the produced sponge was cut in dimensions of 10 × 10 × 10 mm. The weight of each sample was recorded (W_0_). Each sample was then placed separately in 50 mL balloons containing 25 mL of whole human blood (freshly prepared from a healthy volunteer), which was poured on them. After 3 min, the samples were slowly removed from the solution, and then the samples were weighed (W_1_). Blood uptake (W_B_) was calculated from the following formula (grams of blood per gram of substance).

W_B_ = (W_1_ − W_0_)/W_0_

A similar procedure of the blood absorption test was used to measure the swelling percentage of the sponge. The formula below was used to calculate the percentage of swelling:

Swelling percentage = (W_1_ − W_0_)/W _0_ × 100

where W_1_ and W_0_ denote the weight of wet and dry sponges, respectively.

#### 2.4.2. Blood Standard Prothrombin and Partial Thromboplastin Time

Blood coagulation time was assessed for the samples. For this purpose, standard prothrombin time (PT) and partial thromboplastin time (PTT) tests were performed. To prepare human plasma for PT and PTT tests, 1.8 mL of fresh whole blood was prepared from healthy human volunteers and mixed with exactly 0.2 mL of 3.2% sodium citrate and centrifuged at 3000 rpm for 10 min.

To perform the PT test, the first 200 μL of thromboplastin was poured into the tubes, and then all samples with dimensions of 10 × 10 × 10 mm (the produced sponge and the commercial sponges) were thrown into the tubes separately and placed in a 37 °C water bath for 10 min. Then, 100 μL of healthy human plasma was added to the tube, and time was measured by using a chronometer. The contents of the tube were mixed and placed in a 37 ° water bath for 5 s. After this period, the tube was removed, and the tube body dried and was constantly shaken horizontally and vertically; as soon as plasma coagulation was observed, time was stopped and recorded. The obtained time was compared with the standard time of the laboratory and the time table inside the Activity and INR kit. The standard kit time was 13 s.

To perform the PTT test, 100 μL of the plasma sample was added to 100 μL of selenite in a hemolysis tube, and then the samples were thrown into tubes separately and mixed. Afterward, the tubes were placed in 37 °C water bath for 3 min. Then, calcium chloride (100 μL) was added to the above tube and mixed, and a stopwatch was used simultaneously. While the tube was gently shaken every 5 s, after 20 s, the tube came out of the water bath, and as mentioned in the case of PT, the chronometer was stopped as soon as the visualization of the coagulation was obtained. The standard kit time was 40 s.

#### 2.4.3. Blood Clotting Time (BCT)

In separate tubes, samples (the produced sponge) with dimensions of 10 × 10 × 10 mm were thrown. Then, 2 mL of fresh blood was added to the tubes, and the tubes were incubated at 37 °C. Three tubes containing 2 mL of fresh blood were used as controls. A chronometer was used to record clotting times. The tubes were checked in the case of coagulation at different times. The time of complete coagulation was noted. The normal range for blood clotting in this method is 8–16 min.

#### 2.4.4. Blood Clotting Index (BCI)

Samples (the produced sponge) weighing 10 mg were placed in separate tubes, and 200 microliters of freshly citrated blood was poured on them. Only 200 microliters of citrated blood was poured into the other tubes (control). All tubes were placed at 37 °C for 5 min. Then, all were gently added to deionized water (10 mL). Samples were located on a shaker (30 rpm) for 10 min. Then, the absorbance of the samples (for OD_S_ sample and OD_C_ control) was read at 540 nm. The clotting index was obtained from the following equation.

BCI (%) = (OD_S_/OD_C_) × 100

#### 2.4.5. Fibrin Formation Process

A very thin small piece of produced sponge was placed on a glass slide, and a drop of blood was dropped on it and observed under a light microscope at 100×. This was also repeated for blood droplets on a glass slide (without any substance). The results were compared in terms of coagulation times and fibrin formation states.

#### 2.4.6. Platelet Adhesion Test

Platelet-rich plasma was prepared from the blood transfusion organization. Then, 10 mg of the produced sponge was poured into a tube, and 200 microliters of platelet-rich plasma was poured on it and incubated at 37 ° for 10 min. The samples were then washed with PBS and fixed with 5% glutaraldehyde. Glutaraldehyde was then dehydrated using ethanol, and the sample was viewed and imaged under an electron microscope.

#### 2.4.7. Clinical Assessment

After the approval of the ethics committee of Tabriz University of Medical Sciences, 10 patients who required the extraction of two teeth (split-mouth mandibular first molar) were selected. First, each patient announced his consent to participate in the study and completed the written informed consent form. All patients were given chlorhexidine mouthwash before surgery and gelofen after surgery. The extraction of both teeth in all patients was performed by a surgeon under the same conditions.

**Figure 2:**
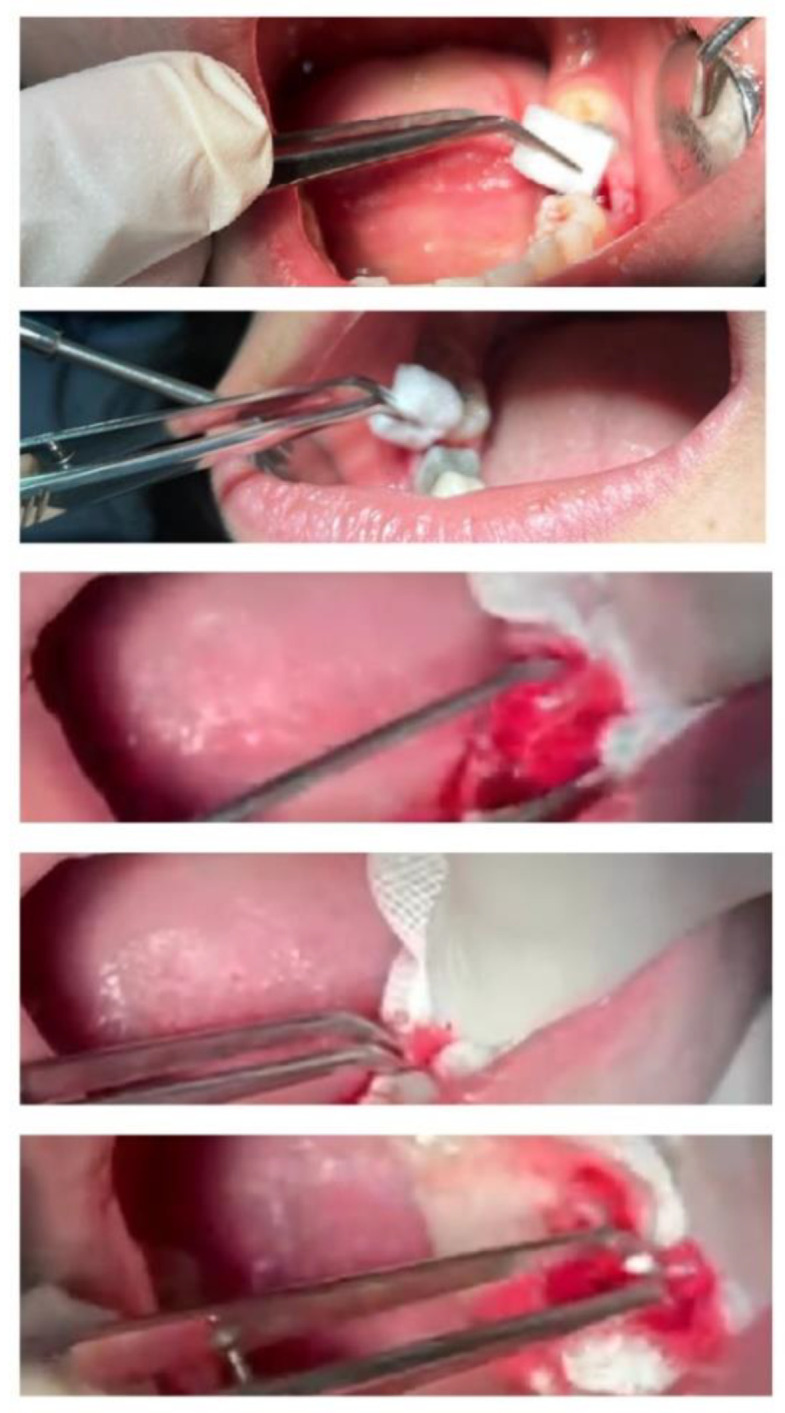
The process for clinical assessment.

After teeth extraction, for dressing, the produced sponge was placed in one of the extracted dental cavities; then, a sterile gauze with a specific weight was placed on it (test group). In the other tooth, for dressing, a commercial sponge (Spongostan^®^, Denmark) was placed in the dental cavity, then a sterile gauze with a certain weight was placed on it (control group). In the studied groups, sterile gas changed every 15 min until bleeding stopped completely. The difference in the weight of sterile gas at the beginning and after blood absorption indicated the amount of blood absorption for each sterile gas. The amount of sterile gas consumed and the total weight of the absorbed blood in each group (test and control) were compared. Figure 2 shows the process.

## 3. Results and Discussion

### 3.1. Physicochemical and Physicomechanical Characterization

#### 3.1.1. Sponge Structure and Morphology

In this study, we used the freeze-drying technique to produce the sponge. Generally, the freeze-drying technique produces sponge-like matrixes. In addition, due to the improvement in stability and rapid solubility in several applications, including drug delivery (e.g., antibiotic and site-specific delivery systems), the freeze-drying technique can be used to produce drug-containing sponges [29,30,31].

**Figure 3:**
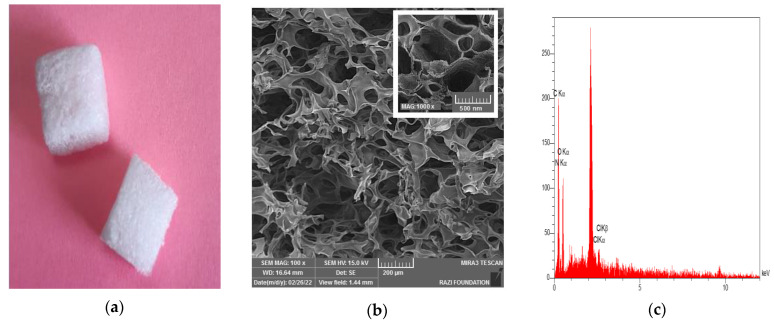
The produced sponge with the dimension of 10 × 10 × 10 mm (**a**). The SEM image of the sponge (**b**) and the EDX analysis (**c**).

Figure 3a shows the produced sponge with a dimension of 10 × 10 × 10 mm. SEM images of the sponge and the EDX analysis are also presented in Figure 3b,c, respectively. The microscopic structure of the prepared sponge under SEM showed a porous structure with micrometric and nanometric pores. Micrometric pores can be used for trapping red blood cells (RBCs), and nanometric pores may help improve water sorption (plasma sorption) [7]. Nanometer-scale pores on the surface of the sponge can also lead to surface roughness [8]. The presence of carbon, nitrogen, and oxygen in the EDX spectra was attributed to gelatin [32].

#### 3.1.2. Identification of the Functional Groups

**Figure 4:**
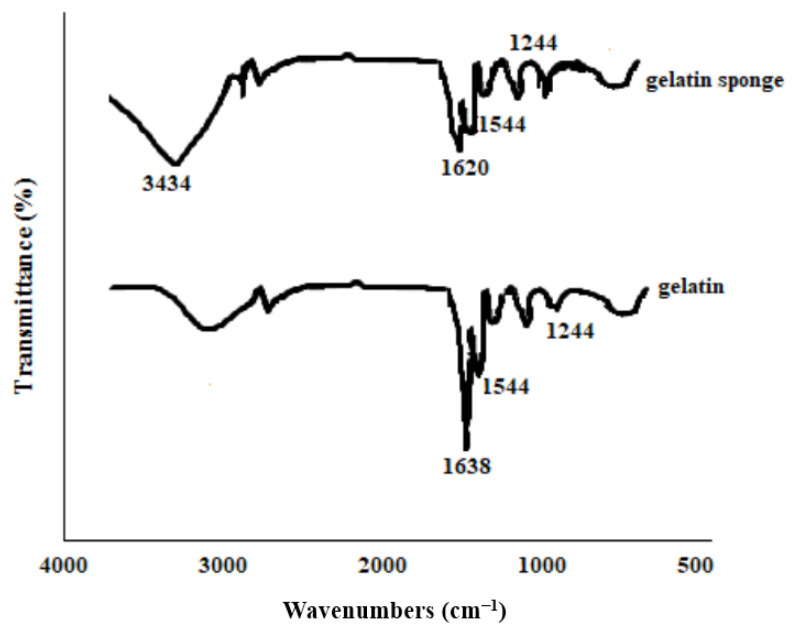
FTIR results for gelatin and the produced sponge.

The FTIR results showed all functional groups of gelatin; 1638 cm^−1^, 1544 cm^−1^, 1244 cm^−1^, and 3433 cm^−1^ represented the amide I, II, and III bands of gelatin, respectively [33,34]. We used glutaraldehyde as a cross-linking agent. Glutaraldehyde is advantageous among other aldehydes used in the protein matrix due to its rapid reaction, availability, low price, high aqueous solubility, and reaction with a considerable number of available amino groups found in the structure of gelatin [35]. The amide I peak for gelatin powder (1638 cm^−1^) in the sponge sample becomes smaller and also had a shift to 1620 cm^−1^. This result implied that gelatin reacted with glutaraldehyde, resulting in the formation of a Schiff base, indicating cross-linking with glutaraldehyde [33,34]. Indeed, the (-CHO) aldehyde group of glutaraldehyde interacts with the lysine amine groups of gelatin proteins [36]. The broad band at 3434 cm^−1^ for the sponge displays the existence of NH stretching, OH stretching, and intermolecular hydrogen bonding [37] (Figure 4).

#### 3.1.3. Identification of Crystallinity State

**Figure 5:**
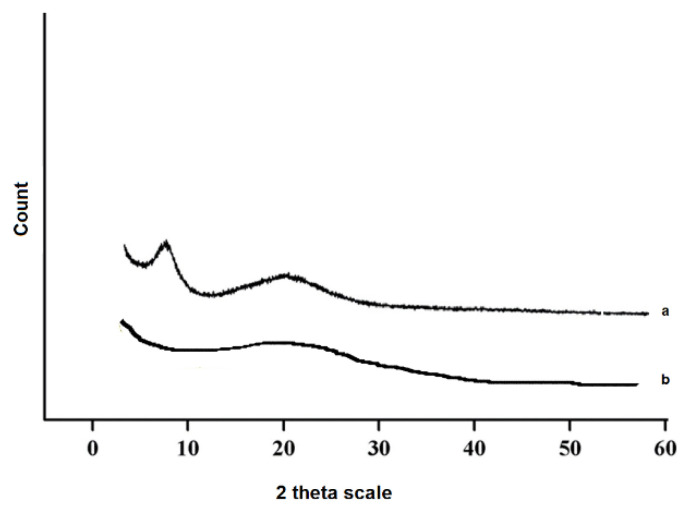
XRD diffraction patterns, gelatin (**a**), and the prepared sponge (**b**).

XRD patterns of gelatin powder (Figure 5a) exhibited two peaks at 2θ around 7° and 20°, corresponding to the content of the triple helix structure of gelatin and the amorphous nature of gelatin, respectively [38]. The peak at 7° considerably disappeared in the XRD peak for the produced sponge (Figure 5b) in the presence of GTA. These outcomes show that the content of the triple helix structure was noticeably disrupted by the addition of GTA. Indeed, the formation of the covalent bond between gelatin and GTA prevents the renaturation of the triple helix structure [39]. Liu et al. reported similar results for gelatin/GTA cross-linked films [39]. According to the literature, 20% content of glycerol in the gelatin solution shows a sharp XRD peak at 2θ around 18° [40]. In this study, the content percentage for glycerol was 1%. Then, it did not show any detectable peak.

#### 3.1.4. Tensile Strength Determination

The strength of surgery gelatin sponges should satisfy their proposed biomedical requests. The produced gelatin sponge has a relatively high tensile strength in a dry state and its elongation at break was 0.093 ± 0.001 MPa. When the material absorbs water, its tensile strength decreases to 0.045 ± 0.003 MPa. Commercial sponges showed a tensile strength of 0.080 ± 0.005 MPa for GELITA-SPON and 0.074 ± 0.008 MPa for Spongostan^®^ in a dry state. The tensile strength of commercial sponges in the wet state was 0.023 ± 0.004 MPa for GELITA-SPON and 0.018 ± 0.010 MPa for Spongostan^®^. The results of tensile strength evaluation show a more flexible structure for the prepared sponge compared to commercial sponges [41]. Using glycerol as a plasticizer and selecting the proper concentration of glutaraldehyde as a cross-linker, the gelatin sponge’s strenghth is while it has no cytotoxicity effects [42,43]. This may provide a means of easily adapting a sponge’s mechanical possessions. These methods can increase the ease of sponge handling during surgical procedures.

#### 3.1.5. The Specific Surface Area, Total Pore Volume, and the Porosity

The ratio of the total pore volume divided by the total volume of sample can be considerd as the porosity percentage of the material. The results showed the porosity percentage of about 63 percent for the produced sponge. Table 2 shows the specific surface area, total pore volume, and the porosity for the produced sponge.

#### 3.1.6. Volume Expansion and Density Determination

Dentists sometimes sterilize the surgical sponge with serum and then insert it into the extracted tooth cavity. From this point of view, it is important to know the volume expansion rate. The volume expansion rate of the produced sponge was 1.31 ± 0.07.

#### 3.1.7. Degradation Test

The application of biodegradable and biocompatible materials that absorb without any problems in the body is one of the important factors [44]. Degradation accounts for the disintegration of the integrity of the material and ultimately the complete dissolution of the sample.

**Figure 6:**
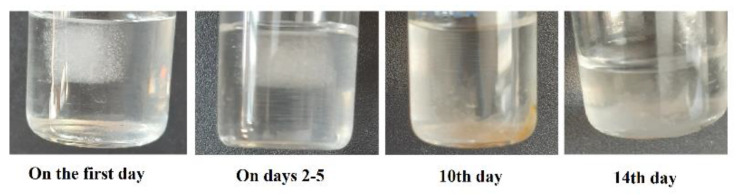
The degradation process of the produced sponge for 2 weeks.

The degradation test of the produced sponge was evaluated for 2 weeks. The results showed that the rate of degradation for the sponge was 14 days. On the first day, the sponge states its cubic form and became a transparent cubic gel. On days 2–5, the sponge’s structure was partially degraded, and it was a transparent gel yet. By increasing the degradation time to 10 days, about 90% of the matrix degraded, and it became a yellow gel. On day 14, no traces of the sponge remained (Figure 6).

### 3.2. Biocompatibility Assay

#### 3.2.1. Cytotoxicity

The study of cytotoxicity for biologically active biomaterials is a required step before in vivo and clinical treatments. For this purpose, the cytotoxicity of the produced sponge was investigated against HFFF2 cell lines by using the MTT method. The produced sponge had no toxic effects on the HFFF2 cells at 3 to 5 days. In addition, abnormal cell morphology was not observed in either the control or test groups. Then, the prepared sponge resulted in good cell compatibility.

#### 3.2.2. Hemolysis Test

It is necessary to examine the compatibility of sponges with RBCs for the safe application of sponges. Checking the ratio of hemolysis is the best technique to evaluate the biocompatibility of the newly prepared sponge with RBCs. This examination was performed according to standard ISO 10993-4, in which the below 2% hemolysis is considered as substance biocompatibility [45,46]. To investigate hemolytic effects, the lysis rates of RBCs were evaluated in the exposure to sponges. The results showed that hemolysis rates of produced sponge, positive control (water), and negative control (PBS) were 0.176 ± 0.008, 100, and 0, respectively.

**Figure 7:**
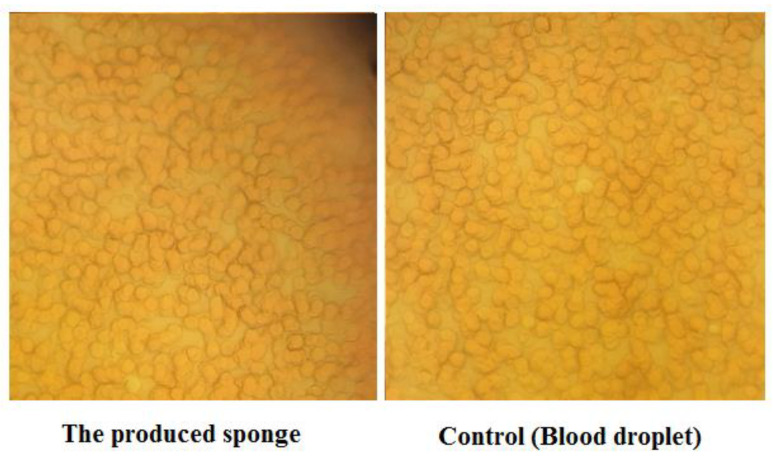
The morphology of RBCs under the light microscope (100×).

The morphology of RBCs was also checked under the light microscope (100×) (Figure 7), showing no toxic effects of the produced sponge on the morphology of RBCs compared to the blood droplet.

In a report by Xi et al., the hemolysis ratio of the polysaccharide-based sponge was 1% [47]. Fang et al. also reported that the hemolysis ratio of their prepared sponge (chitin) was 2.5% [48]. In another study, a chitosan-based sponge showed a hemolysis ratio of 3.5% [49]. Then, according to our results, the prepared sponge displayed very good hemo-biocompatibility.

### 3.3. Function Evaluation Tests

#### 3.3.1. Blood Absorption and Swelling Percentage

The produced sponge showed the ability to absorb blood 35 times its weight. It can be noted that the prepared sponge traps blood cells due to its high porosity (the microporous structure that matches better with the micrometric size) [50]. Swelling percentage is a specific possession for hydrophilic and porous materials. The produced sponge showed a high swelling percentage of 3500%. Sponges with high blood absorption and swelling percentage can keep the wound’s surface moist and facilitate wound healing [51,52]. It is also reported that high swelling capacities can enhance the hemostasis of sponges by concentrating clotting factors within the sample [51]. The other investigators also reported porous sponges with the ability to absorb blood 10 times its weight (chitosan sponge) [53] and 60 times its weight for a cellulose-based superabsorbent hemostatic agent [54]. However, these were not dental surgery sponges. There were no reports about dental-sponge surgery foams to compare with our results. Moreover, in some studies, the absorption capacity of a hemostatic material was studied with water, PBS, or simulated body fluid absorption instead of a whole blood sample [47,48,55].

#### 3.3.2. Blood Standard Prothrombin and Partial Thromboplastin Time

The PT and PTT results of the produced sponge were 12.91 ± 0.16 and 39.49 ± 0.62, respectively. All samples had PT and PTT times below the standards (the standard kit time was 13 s for PT and 40 s for PTT).

#### 3.3.3. CT Coagulation Time

**Figure 8:**
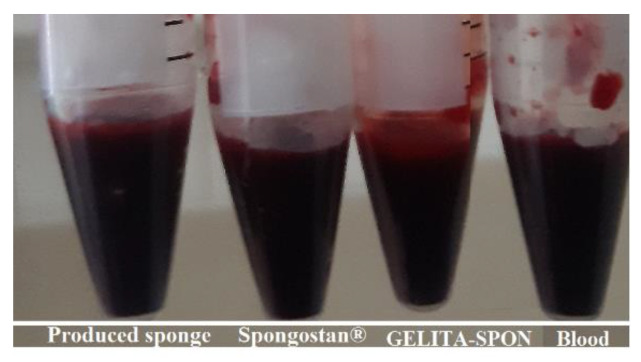
The results for CT coagulation time.

The prepared sponge showed lower CT coagulation times (243.33 ± 2.35 s) compared to commercial dental surgery sponges (362.33 ± 2.05 s for GELITA-SPON and 540 ± 1.63 s for Spongostan^®^), which displays its ability for faster coagulation. Figure 8 shows the results for CT coagulation time. Blood without any sponge (as control) shows coagulation times of 895.33 ± 3.39 s.

#### 3.3.4. Blood Clotting Index (BCI)

**Figure 9:**
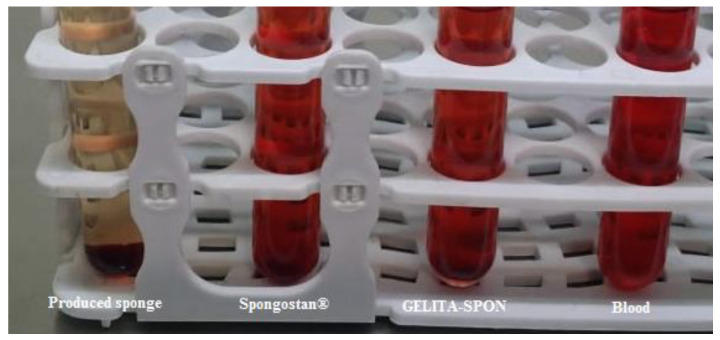
The results for blood clotting index (BCI).

The in vitro hemostatic capability of the produced sponge was tested using a dynamic whole-blood clotting model. The blood clotting index (BCI) evaluates the generation of stable clots by calculating the absorption spectrum of hemoglobin liberated from non-attached RBCs. The produced absorbent with a low-value test shows the desired hemostatic function [47]. As illustrated in Figure 9, the constructed sponge exhibits improved performances compared to commercial ones. The produced sponge exhibits a BCI of 10.67 ± 0.004% while the commercial ones show a BCI of 75.19 ± 0.028% for GELITA-SPON and 85.15 ± 0.038% for Spongostan^®^, respectively. Blood without any sponge (as control) showed a BCI of 100 ± 0%. Wang et al. developed porous carboxymethyl cellulose hemostatic agents with an MCI value of 14–29 percent [56]. Moreover, Xie et al. fabricated a polysaccharide-based sponge with a BCI of 5–10% [47], while this amount was 3–25% for a chitosan sponge [49]. Collectively, the porous structure is one of the main reasons for low BCI levels [48].

#### 3.3.5. Platelet Adhesion Test

**Figure 10:**
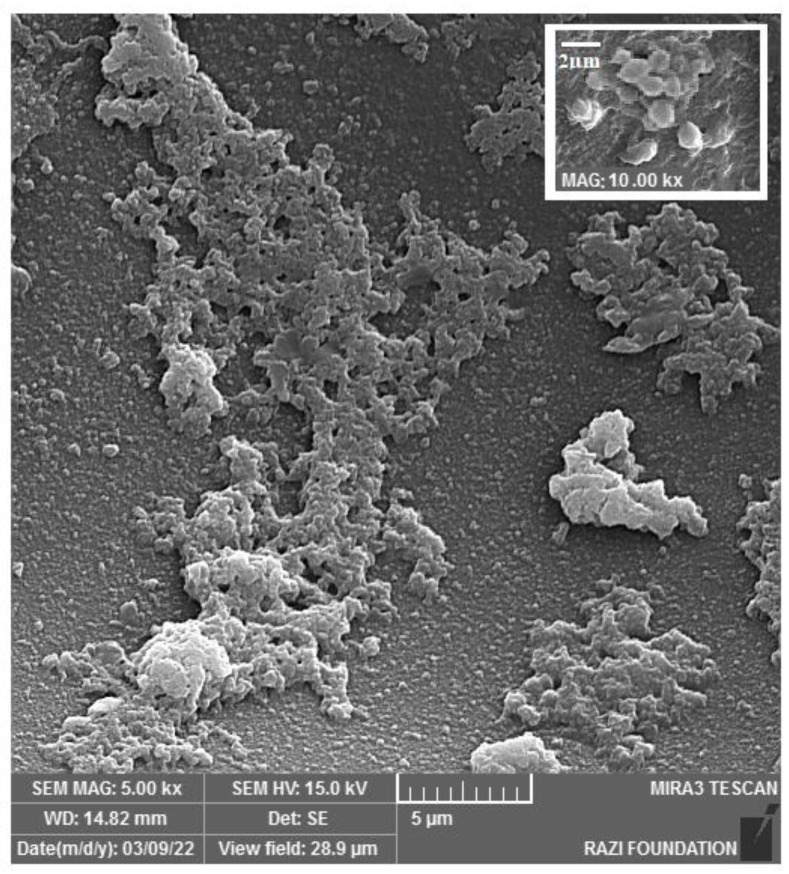
The absorption and aggregation of platelets on the produced sponge.

The aggregation of the platelets at the bleeding area that stimulates the coagulation process occurs as it is the main step of bleeding control [57]. Thus, the capability of the produced sponge in inducing the aggregation and accumulation of platelets was investigated by analyzing SEM images. As shown in Figure 10, the sponge exhibited a high potency in the absorption and aggregation of platelets. The sponge contains both micrometric and nanometric pores. Nanometric pores absorb the blood plasma, while the platelets and RBCs are trapped in micrometric pores, resulting in the activation of hemostasis [47].

#### 3.3.6. Fibrin Formation Process

**Figure 11:**
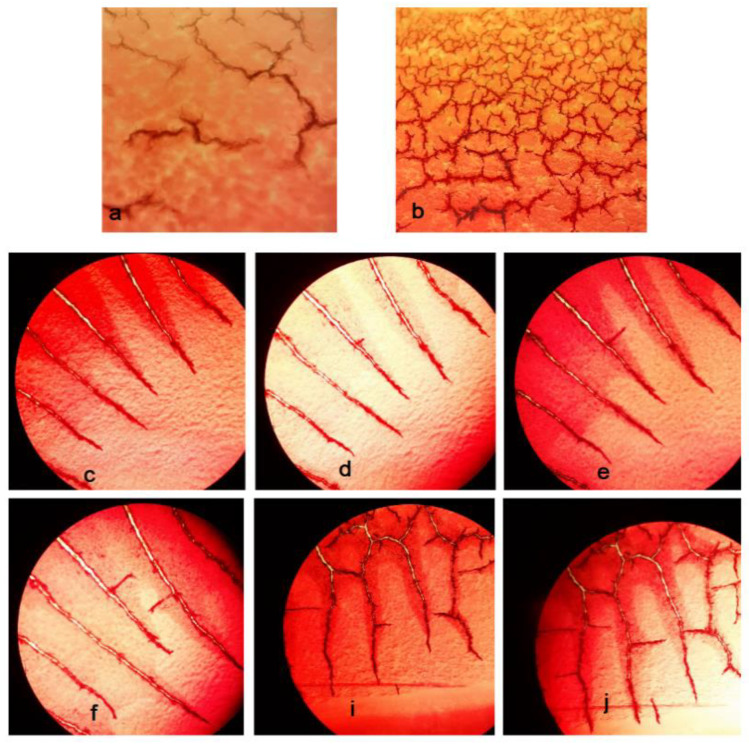
The time points of the fibrin formation process under a light microscope (100×): (**a**) on the production sponge at the time of 30 s, (**b**) on the production sponge at the time of 70 s, and (**c**–**j**) related to the blood droplet from 180 to 240 s.

The gelatin found in the structure of the sponge triggers the process of hemostasis by stimulating platelet activations and the initiation of coagulation [58]. The aggravation of platelets in the coagulation cascade is followed by the polymerization of the fibrin chains [59]. The fibrin formation process on the production sponge was initiated at the time of 30 s and continued until 70 s. Fibrin formation on the sponge was rapid with the formation of various fibrin networks throughout the sponge. The fibrin formation process related to the blood droplet initiated at the time of 180 s and continued until 240 s. The process started from one side and continued to the other side of the blood droplet. Figure 11 shows the time points of the fibrin formation process under the production sponge (Figure 11a,b) and the blood droplet (Figure 11c–j).

#### 3.3.7. Clinical Assessment

The use of absorbable gelatin sponges has been reported as progress in oral surgery. Postoperative problems and the need for extended treatment are often evaded by sponges’ use [19,60]. Table 3 shows the results of clinical assessments, including the number of used sterile gauze and the total weight of the absorbed blood. The results showed no significant differences between the number of used gauzes for the test and control groups. However, the total weight of absorbed blood (by sterile gauze) showed significant differences (*p*-value = 0.0015). The total weight of absorbed blood by a sterile gauze was higher in the control group, showing the superior ability for blood absorption with respect to the test sponge. Walter et al. collaborated in gathering data on a series of 250 cases in which an absorbable gelatin sponge (Gelfoam) was employed. They concluded that absorbable gelatin sponges are good for assistance in oral surgery. They reported that the significance of the absorbable sponge is dualistic: first, as a hemostatic agent, and second, to eliminate “dead space” [19].

## 4. Conclusions

The produced sponge in this study showed great abilities in bleeding control and no toxic effects with respect to HFFF2 cells and RBCs. It showed a porous structure with micrometric and nanometric pores that is beneficial for the simultaneous trapping of RBCs and improved plasma sorption. It had a flexible nature that eased its handling during the surgery process. These benefits may help enhance functions in the surgical application of sponges. According to the clinical results, the total weight of blood absorbed by the sterile gauze showed significant differences, and the total weight of absorbed blood was higher in the control group, showing the superior ability of blood absorption for the produced sponge. The sponge is recommended for use in dental surgeries because of its abilities in bleeding control.

## Figures and Tables

**Table 2:** The specific surface area, total pore volume, and the porosity for the produced sponge.

Specific Surface (m^2^/g)	Pore Radius (nm)	Total Pore Volume (cm^3^)	Porosity (%)
5.3	120	0.63	63

**Table 3:** The results of clinical assessments include the number of used sterile gas and the total weight of absorbed blood.

Group	The Number of Used Sterile Gauze (Mean Number)	The Total Weight of Absorbed Blood by Sterile Gauze (g/g)
**Test**	2.4 ± 0.48	2.67 ± 1.17
**Control**	2.6 ± 0.48	4.27 ± 0.49
***t*** **-test *p*-value**	0.39	0.0015

## Data Availability

The raw/processed data can be shared by request from the corresponding author.
